# Effect of Sintilimab combined with Chemotherapy on Tumor Markers and Immune Function of advanced non-small cell lung cancer

**DOI:** 10.12669/pjms.37.4.3820

**Published:** 2021

**Authors:** Xiaoyan Liang, Zhangfeng Wei

**Affiliations:** 1 Xiaoyan Liang, Collage of Pharmacy, Xi’an Medical University, Xi’an, Shaanxi 710021, P.R. China; 2 Zhangfeng Wei, Cardio-Thoracic Surgery, Xishan Coal Electricity Group Worker General Hospital, Taiyuan, Shanxi 030053, P.R. China

**Keywords:** Chemotherapy, Immune function, Non-small cell lung cancer, Sintilimab, Tumor markers

## Abstract

**Objective:**

To evaluate the effect of sintilimab combined with chemotherapy on tumor markers and immune function in advanced non-small cell lung cancer.

**Methods:**

The study was conducted at Xi’an Medical University, China. The 120 patients with advanced NSCLC who were treated in our hospital from January 2016 to January 2020 were randomly divided into two groups, with 60 cases in each group. Patients in the control group received conventional GP chemotherapy, while those in the experimental group received intravenous injection of sindilimab on the basis of conventional GP chemotherapy. The changes of serum tumor markers CYFRA211, CEA, CA125 and T lymphocyte subsets CD3+, CD4+, CD8+, CD4+/CD8+ in the two groups prior to and after treatment were compared and analyzed. At the same time, the clinical efficacy at six months was compared between the two groups.

**Results:**

The serum tumor markers CYFRA211, CEA and CA125 in the two groups after treatment were lower than those before treatment, and the difference was statistically significant (P=0.00). Specifically, the above-mentioned markers in the experimental group decreased more significantly than those in the control group, and the difference was statistically significant (CYFRA211, CA125, p=0.00; CEA, p=0.01; the levels of CD3+ and CD4+ in the experimental group were higher than those in the control group after treatment, with statistical significance (CD3+, p=0.00; CD4+, p=0.01)). No significant change can be seen in CD8+ (p=0.14), and the level of CD4+/CD8+ in the experimental group was higher than that in the control group, with a significant difference (p=0.02). The complete remission rate (CR) was 22% in the experimental group and 8% in the control group (P=0.04), which was statistically significant. The progress rate (PD) of the experimental group was significantly lower than that of the control group, with statistical significance (p=0.02). The overall response rate (RR) of the experimental group was more advantageous than that of the control group, with a statistically significant difference (p=0.01).

**Conclusion:**

Compared with chemotherapy alone, significant therapeutic effects can be obtained in the treatment of advanced non-small cell lung cancer with sintilimab combined with chemotherapy. With this combination regimen, the level of serum tumor markers can be significantly reduced, the cellular immune function of patients can be improved, with the overall response rate of treatment increased, and the risk of progressive disease of patients reduced.

## INTRODUCTION

Lung cancer, in terms of clinical practice, is one of the most common malignant tumors of the respiratory system, with a high mortality rate. Most of the pathological types of lung cancer are non-small cell lung cancer (NSCLC), accounting for more than 80% of the total incidence of lung cancer.^1^ Patients with advanced NSCLC are mainly treated with chemotherapy. However, patients vary in their response to chemotherapy drugs and side effects, so the therapeutic effect and safety are significantly different.^2^ With the continuous progress of gene technology, benefits are obtained from targeted drugs by an increasing number of patients with advanced NSCLC.^3^ Certain gene tests, such as XPG gene testing, may be used as predictors of individualized NSCLC treatment strategies.^4^ Currently, PD-1/PDL-1 monoclonal antibody is widely used in the treatment of lung cancer. Sintilimab, as a PD-1 monoclonal antibody, can bind to PD-1 on the surface of T cells and block the binding between it and the ligand PD-L1, so that T cells and autoimmune reactions can play a normal role, thereby eliminating Tumor cell.^5^ This study evaluated the efficacy of sintilimumab combined with chemotherapy on advanced NSCLC by recording tumor markers and immune function before and after treatment.

## METHODS

### Ethical approval

The study was approved by the Institutional Ethics Committee of Xi’an Medical University, (dated September 21, 2020) and written informed consent was obtained from all participants.

### Inclusion criteria:

1. The diagnostic criteria of advanced NSCLC are met.^6^
2. The patient has no obvious cognitive impairment and agrees with the study protocol and signs the consent form.

### Exclusion criteria:

1. Severe basic diseases such as diabetes, cardiovascular and cerebrovascular diseases.
2. Mental disorders, unable to cooperate to complete the study.
3. Complicated diseases affecting the level of tumor markers such as other malignant tumors.
4. Complicated diseases affecting the level of immune factors such as chronic inflammatory diseases and autoimmune diseases.

The 120 patients with advanced NSCLC who were treated in our hospital from January 2016 to January 2020 were randomly divided into two groups, with 60 cases in each group. Among them, 31 males and 29 females were enrolled to the experimental group, with an average age of 66.38 ± 6.87, ranging from 53 to 76 years old, 33 males and 27 females were enrolled to the control group, aged 51-76 years, with an average of 63.15±8.70, ranging from 51 to 76 years old. ECOG score of all patients was ≤ 2, and the physical status was suitable for chemotherapy. There was no significant difference in the general information between the two groups, and the groups were comparable (Table-I).

**Table-I:** Comparative analysis of general data of the two groups of patients (*X̅* ±S) n=30.

	Experimental group	Control group	t/χ^2^	p
Male (%)	31(52%)	33(55%)	0.28	0.60
Age (years)	66.38±6.87	63.15±8.70	1.60	0.12
Weight (kg)	67.07±10.20	65.53±11.13	0.56	0.58
ECOG scores	0.69±0.23	0.72±0.25	0.48	0.63

p>0.05

### Therapeutic methods

Patients in the control group were given conventional GP chemotherapy:^6^ Prior to chemotherapy, routine biochemical and blood routine examinations were performed. Patients with a significant decrease in blood white blood cells were treated with drugs for increasing white blood cells. Hydration was carried out one day before chemotherapy, gemcitabine (1 g/m2) was intravenously on the 1st and 8th day, and cisplatin (25 mg/m2) was intravenously on the 1st to 3rd day. During the chemotherapy, all patients received symptomatic treatment, such as electrocardiogram monitoring, hydration, and treatment for adverse reactions. The chemotherapy was repeated every 21 days for three cycles.^7^ Patients in the experimental group were treated with sindilimab on the basis of chemotherapy: intravenous infusion of 200mg sindilimab, once every 3 weeks.^8^

### Adverse drug reactions and treatment

The most common adverse reactions during treatment include nausea and vomiting and gastric ulcer. We take advantage of hydration, and also use acid suppressants such as omeprazole and gastric mucosal protective agents represented by gastricin to prevent acid suppression and antiemetics. For acute vomiting, we use dexamethasone or tropisetron.

### Observation indexes

Prior to treatment and after the treatment cycle, the morning and fasting serum was sampled to compare and analyze the changes of serum tumor markers CYFRA211, CEA and CA125 and the levels of CD3+, CD4+, CD8+ and CD4+/CD8+ in T lymphocyte subsets between the two groups. The clinical efficacy (short-term efficacy) of the two groups at six months was compared and analyzed. Methods of clinical efficacy judgment^9^: complete remission (CR): the lesion completely disappeared, and the tumor marker detection results returned to normal, and maintained for more than four weeks; Partial response (PR): the lesion volume decreased by more than 30%, and maintained for more than four weeks; Stable disease (SD): the volume of lesions decreased by < 30% or increased < 30%; Progressive disease (PD): the volume of lesions increased by more than 30% or new lesions appeared; overall response rate (RR)=CR + PR.

### Statistical analysis

All the data were statistically analyzed with SPSS 20.0 software, and the measurement data were expressed as (*X¯*± S). Two independent sample t-test was used for inter group data analysis, paired t-test for intra group data analysis, and χ^2^ test for rate comparison. P<0.05 indicates a statistically significant difference.

## RESULTS

The changes of serum tumor markers in the two groups prior to and after treatment are shown in Table-II. As suggested by this study, the CCYFRA211, CEA, CA125 and other indicators in the experimental group and the control group after treatment were lower than those before treatment, with a statistically significant difference (P=0.00). The experimental group showed a more significant reduction than the control group, and the difference was statistically significant (CYFRA211, CA125, p=0.00; CEA, p=0.01).

**Table-II:** Comparative analysis of serum tumor markers prior to and after treatment (*X̅* ± S) n=60

Observation indexes	CYFRA211(ng/ml)				CEA(ng/ml)				CA125(U/ml)			

Groups	Prior to treatment*	After treatmentΔ	t	p	Prior to treatment*	After treatmentΔ	t	p	Prior to treatment*	After treatmentΔ	t	p
Experimental group Δ	25.34±5.31	6.97±0.54	19.86	0.00	13.44±5.23	4.63±2.41	8.38	0.00	147.74±14.35	28.37±5.24	42.58	0.00
Control group Δ	23.73±4.66	11.23±4.72	10.32	0.00	13.29±6.10	7.25±2.32	5.07	0.00	149.13±15.24	53.72±8.23	30.17	0.00
t	1.24	4.91			0.10	4.28			0.36	14.23		
p	0.22	0.00			0.92	0.01			0.74	0.00		

* p>0.05, Δp<0.05

As indicated by the changes of T cell subsets in the two groups prior to and after treatment. Table-III. The levels of CD3+ and CD4+ in the experimental group were higher than those in the control group, with statistical significance (CD3+, p=0.00; CD4+, p=0.01). No significant changes can be seen in CD8+ (p=0.14). The level of CD4+/CD8+ in the experimental group was higher than that in the control group, with significant difference (p=0.02).

**Table-III:** Comparative analysis of serum tumor markers prior to and after treatment (*X̅* S) n=60

Observation indexes	CD3^+^(%)		CD4^+^(%)		CD8^+^(%)		CD4^+^/CD8^+^(%)	

Groups	Prior to treatment	After treatment*	Prior to treatment	After treatment*	Prior to treatment	After treatment	Prior to treatment	After treatment*
Experimental group	60.71±8.52	77.34±9.36	33.65±5.32	37.95±6.02	24.47±3.97	23.72±4.18	1.77±0.76	1.92±0.48
Control group	62.38±7.73	60.21±5.48	33.58±4.75	31.36±5.94	24.25±3.04	25.15±3.37	1.73±0.58	1.42±0.53
χ^2^/t	0.80	8.65	0.05	4.27	0.24	1.47	0.23	3.83
p	0.43	0.00	0.96	0.01	0.81	0.14	0.82	0.02

* p<0.05

As indicated by the comparison of clinical efficacy between the two groups of patients prior to and after treatment (Table-IV): CR was 22% in the experimental group and 8% in the control group, which was statistically different (p=0.04); PD in the experimental group was significantly lower than that in the control group, with statistical significance (p=0.02); The overall response rate (RR) of the experimental group was more advantageous than that of the control group, with a statistically significant difference (p=0.01).

**Table-IV:** Comparative analysis of clinical efficacy of two groups prior to and after treatment (*X̅* S) n=60

Indexes	CR (%)*	RR (%)	SD (%)	PD (%)*	RR (%)*
Experimental group	13(22%)	25(42%)	17(28%)	5(8%)	38(64)
Control group	5(8%)	20(33%)	25(42%)	10(17%)	25(41%)
χ^2^	4.18	4.88	2.34	4.18	5.65
p	0.04	0.34	0.12	0.02	0.01

* p<0.05.

***Notes:*** CR=complete remission; PR=partial remission; SD=stable disease; PD=progressive disease; RR=overall response rate; RR=CR + PR.

## DISCUSSION

Lung cancer, is the most common malignant tumor of the respiratory system, as its pathological type mainly NSCLC. Chemotherapy, targeted therapy and immunotherapy are the main treatment options for advanced NSCLC. For some patients with advanced NSCLC, the prognosis and survival rate can be improved by chemotherapy, but obvious adverse reactions may also occur.^10^ The combination therapy, however, is superior to chemotherapy alone, with the advantages of high safety and low side effects.^11^ In recent years, with the role of the immune mechanism in tumor formation and development has been continuously clarified, new immunotherapy targets are also gradually clear. It is reckoned in some studies that.^12^ in the process of tumor formation and progression, cellular immune deficiency may cause tumor cells to evade the immune system, and then escape, leading to the reduction or even disappearance of the therapeutic effect of anti-tumor drugs.

Presently, targeted drugs and immune drugs are recommended by an increasing number of lung cancer diagnosis and treatment guidelines for the treatment of patients with advanced NSCLC.^13^,^14^ Different from targeted drugs directly attacking tumor cell targets, the main purpose of immunotherapy is to regulate the autoimmune state of patients to remove tumor cells. PD-1 is mainly expressed on the surface of immune cells, while PDL-1 on the surface of tumor cells. The combination of PD-1 and PDL-1 contributes to immune escape of tumor cells by activating the signal pathway in immune cells. Studies by He et al. suggest that PD-1 can inhibit cellular immunity, and the killing effect of TIL can be increased by blocking PD-1/PD-L1.^15^ Whether PD-L1 is positive in tumor micro-environment can be used as an indicator to judge the effect of immunotherapy.^16^

The overall survival rate of patients with metastatic NSCLC has been improved by biomarker-oriented targeted therapy and immunotherapy.^17^ As an immunotherapeutic drug, PD-1 monoclonal antibody has been approved by the National Food and Drug Administration (FDA) for clinical treatment of patients with NSCLC.^18^ It restores the immune function of cells by specifically blocking the PD-1 inhibition pathway, and then suppresses tumor cells.^19^ As indicated by the results of a multicenter study.^20^ Only about 20% of patients with chemotherapy combined with immunotherapy withdrew from the study due to adverse reactions, while most patients were able to complete the whole course of treatment and achieve good results, with an overall response rate of 70%. In addition, a study conducted by Chen H et al.^21^ also confirmed that significant clinical benefits were achieved by immune checkpoint blockade (ICB) in the treatment of NSCLC. It can be seen from the results of our study that: after treatment, the CD3+ and CD4+ levels of T cell subsets in patients receiving sintilimab combined with chemotherapy were higher than those in the chemotherapy group alone, which was statistically significant (CD3+, p=0.00; CD4+, p=0.01). The level of CD4+/CD8+ in patients receiving sintilimab combined with chemotherapy was higher than that in the control group (p=0.02). The overall response rate (RR) of sintilimab combined with chemotherapy group was 64%, which was significantly higher than that of the control group, and the difference between the two groups was statistically significant (p=0.01). It is confirmed that PD-1 monoclonal antibody is conducive to improving and regulating cellular immune function, exerting a role in killing tumor, inhibiting disease progression, and satisfactory clinical benefits can be obtained on this basis. However, the overall response rate was lower than that reported in the literature (64% vs. 70%), which may be linked to the fact that all the patients involved in the study were patients with advanced NSCLC.

Early diagnosis is of great significance for patients with lung cancer, especially for those with mild or no history of tobacco use, early detection of tumor markers is essential.^22^ Studies have confirmed that CEA combined with CYFRA21-1 contributes to the auxiliary diagnosis of LAC, and CA125 is linked to metastasis.^23^ The decrease of tumor markers also indicates the effectiveness of treatment and good prognosis of patients.^24^ This study confirmed that the level of tumor markers in the experimental group decreased more significantly than the control group after treatment (CYFRA211, CA125, p=0.00; CEA, p=0.01), indicating that sintilimab combined with chemotherapy has obvious advantages over chemotherapy alone.

### Limitations of the study

In terms of the shortcomings of this study, only the clinical efficacy of the two treatment schemes was compared and analyzed. In view of the cumbersome classification of adverse reactions of chemotherapy drugs and PD-1 monoclonal antibody, as well as the lack of a unified classification scheme of related adverse reactions, there is no comparative analysis on the side effects and adverse reactions of the two groups of patients, so as to further confirm the feasibility of the treatment scheme. In response to this, cases and related experience are being actively accumulated by us in order to lay the foundation for further research.

## CONCLUSIONS

Compared with chemotherapy alone, significant therapeutic effects can be obtained in the treatment of advanced NSCLC with sintilimab combined with chemotherapy. With this combination regimen, the level of serum tumor markers can be significantly reduced, the cellular immune function of patients can be improved, with the overall response rate of treatment increased, and the risk of progressive disease of patients reduced.

### Authors’ Contributions:

**XL** designed this study and prepared this manuscript, and is responsible and accountable for the accuracy or integrity of the work.

**XL and ZW** collected and analyzed clinical data.

**ZW** significantly revised this manuscript.
